# Combined therapy with oncolytic adenoviruses encoding TRAIL and IL-12 genes markedly suppressed human hepatocellular carcinoma both in vitro and in an orthotopic transplanted mouse model

**DOI:** 10.1186/s13046-016-0353-8

**Published:** 2016-05-06

**Authors:** Adel Galal El-Shemi, Ahmad Mohammed Ashshi, Youjin Na, Yan Li, Mohammed Basalamah, Faisal Ahmad Al-Allaf, Eonju Oh, Bo-Kyeong Jung, Chae-Ok YUN

**Affiliations:** Department of Laboratory Medicine, Faculty of Applied Medical Sciences, Umm Al-Qura University, PO Box 7607, Holy Makkah, Saudi Arabia; Department of Bioengineering, College of Engineering, Hanyang University, 222 Wangsinmi-ro, Seongdong-gu Seoul, Korea; Graduate Program for Nanomedical Science, Yonsei University, Seoul, Korea; Department of Pathology, Faculty of Medicine, Umm Al-Qura University, Holy Makkah, Saudi Arabia; Science and Technology Unit & Department of Medical Genetics, Faculty of Medicine, Umm Al-Qura University, Holy Makkah, Saudi Arabia; Department of Pharmacology, Faculty of Medicine, Assiut University, Assiut, Egypt

**Keywords:** Dual gene virotherapy, Oncolytic adenoviruses, Interleukin-12, Tumor necrosis factor-related apoptosis-inducing ligand (TRAIL), Hepatocellular carcinoma

## Abstract

**Background:**

Gene-based virotherapy mediated by oncolytic viruses is currently experiencing a renaissance in cancer therapy. However, relatively little attention has been given to the potentiality of dual gene virotherapy strategy as a novel therapeutic approach to mediate triplex anticancer combination effects, particularly if the two suitable genes are well chosen. Both tumor necrosis factor-related apoptosis-inducing ligand (TRAIL) and interleukin-12 (IL-12) have been emerged as promising pharmacological candidates in cancer therapy; however, the combined efficacy of TRAIL and IL-12 genes for treatment of human hepatocellular carcinoma (HCC) remains to be determined.

**Methods:**

Herein, we investigated the therapeutic efficacy of concurrent therapy with two armed oncolytic adenoviruses encoding human TRAIL gene (Ad-ΔB/TRAIL) and IL-12 gene (Ad-ΔB/IL-12), respectively, on preclinical models of human HCC, and also elucidated the possible underlying mechanisms. The effects of Ad-ΔB/TRAIL+Ad-ΔB/IL-12 combination therapy were assessed both in vitro on Hep3B and HuH7 human HCC cell lines and in vivo on HCC-orthotopic model established in the livers of athymic nude mice by intrahepatic implantation of human Hep3B cells.

**Results:**

Compared to therapy with non-armed control Ad-ΔB, combined therapy with Ad-ΔB/TRAIL+Ad-ΔB/IL-12 elicited profound anti-HCC killing effects on Hep3B and HuH7 cells and on the transplanted Hep3B-orthotopic model. Efficient viral replication and TRAIL and IL-12 expression were also confirmed in HCC cells and the harvested tumor tissues treated with this combination therapy. Mechanistically, co-therapy with Ad-ΔB/TRAIL+Ad-ΔB/IL-12 exhibited an enhanced effect on apoptosis promotion, activation of caspase-3 and-8, generation of anti-tumor immune response evidenced by upregulation of interferon gamma (IFN-γ) production and infiltration of natural killer-and antigen presenting cells, and remarkable repression of intratumor vascular endothelial growth factor (VEGF) and cluster of differentiation 31 (CD31) expression and tumor microvessel density.

**Conclusions:**

Overall, our data showed a favorable therapeutic effect of Ad-ΔB/TRAIL+Ad-ΔB/IL-12 combination therapy against human HCC, and may therefore constitute a promising and effective therapeutic strategy for treating human HCC. However, further studies are warranted for its reliable clinical translation.

**Electronic supplementary material:**

The online version of this article (doi:10.1186/s13046-016-0353-8) contains supplementary material, which is available to authorized users.

## Background

Hepatocellular carcinoma (HCC), the 3rd leading cause of cancer-related deaths globally [1, 2], is an aggressive primary liver cancer that its medical therapy represents an important challenge and area of active investigation [3]. In this regard, fewer than 25 % of HCC patients are candidates for curative surgical resection or transplantation, and the earlier therapy with standard chemotherapeutic drugs such as doxorubicin and cisplatin exhibited only low objective response rates (typically <10 %) and accompanied with high toxicity side effects [2, 4]. Furthermore, despite the clinical approval of new drugs with more specific targets such as sorafenib and bevacizumab for treatment of advanced HCC, their overall therapeutic outcome and survival benefit remains poor and haven’t shown any imposing results, and they are also associated with significant toxicity [2, 4]. Therefore, there is a paramount medical demand to explore new therapeutic strategies to improve the management of HCC. To that end, cancer gene therapy coupled with therapeutically useful oncolytic viruses (OVs), including oncolytic adenoviruses (OAds), is being the most attractive therapeutic approach in this setting [5–7].

Over the past two decades, gene therapy, mediated by viral and non-viral vectors, has been developed as a promising pharmacological approach to provide potential treatment options for a variety of cancers and other dark diseases that would otherwise be considered untreatable by conventional drugs [7]. Furthermore, utilization of cancer virotherapy by using replication-competent OAds and other OVs has recently emerged as a novel therapeutic tool; and this premise lies in their preferential and selective replication in cancer cells while sparing normal cells [5–11]. Interestingly, clinical trials using OAd have achieved an obvious anti-cancer effect in patients with head and neck cancer [7]; and to date, hundreds of HCC patients have been treated with this strategy in phase 1 and II clinical trials with encouraging emerging data [6]. However, a single treatment modality may not be sufficient to achieve satisfactory anticancer effect, because most cancers arise from abnormalities in multiple genetic and signal transduction pathways and have complexity and heterogeneity of tumor cells growth, progression and metastasis [12]. Therefore, application of combined therapeutic approaches with different mechanisms to affect multiple tumorigenesis and cancerous pathways has been suggested as an important tactic for effective clinical cancer therapy. Toward this goal, many trials based on arming of OAds with an immuno-stimulatory-, a pro-apoptotic- or another anti-oncogene or tumor suppressor gene were conceived in various studies and have shown improved their antitumor efficacies [5, 7–10]. By far, recent modification of this strategy to cancer targeting dual gene virotherapy; in which OAds have been utilized to simultaneously express dual anticancer genes, have attracted a further deal of interest in providing more satisfactory multimodal cancer killing mechanisms through the selective viral lytic effect on cancer cells and the additive or synergetic interaction between the two expressed anti-cancer genes [13–15].

Human HCC has a notorious resistant to apoptosis induced by conventional chemotherapeutic drugs and thereby development of new modalities to trigger apoptosis in HCC may result in new effective therapeutic tools [12, 16]. In that respect, TRAIL has been proved as a safe and a promising agent for apoptosis-mediated therapy, whereby it offers a fulminant apoptotic effect in various human cancer cell types [17–19], including HCC [20], while showing only negligible effects on normal cells including normal liver cells [17–20]. With respect to the liver, TRAIL has also served as a new therapy for liver fibrosis/cirrhosis in addition to its unique tumouricidal properties [21]. Despite these advantages, earlier clinical trials using recombinant TRAIL or soluble TRAIL receptor-agonists for treatment of cancer patients have been halted due to very short blood circulation half-life and poor pharmacokinetic property; indicating that TRAIL monotherapy is not sufficient [16, 18, 19, 21]; and suggesting the importance of TRAIL combination therapy with other potential anticancer agents/modalities to restore TRAIL-apoptotic functionality and to improve overall tumouricidal response [16]. In support, OAd-mediated combined gene delivery of TRAIL with another antitumor gene has resulted in a more potent antitumor effect than OAd-mediated TRAIL gene delivery alone [13, 14].

In a constant line, cytokine-based cancer immunotherapy has been pursued in a number of ways and now is becoming a clinical reality. In that respect, IL-12, a Th1-cytokine, boosts a potent ability to induce antitumor-specific immunity through pleiotropic effects on natural killer (NK)-and other different immune cells; suggesting its potentiality in treating advanced cancer [22–24]. IL-12 has also shown to have antiangiogenic property and remodeling effect on the peri-tumor extracellular matrix and tumor stroma [23]. Nevertheless, like TRAIL, the robust antitumor activities exerted by using human recombinant IL-12 has not yet been successfully translated and sustained into the clinics due to its poor pharmacokinetics associated with shortening antitumor efficacy [23]. The logical consequence of these setbacks was the commencements of development of IL-12-based gene therapy to not only optimize IL-12′s *in situ* expression in the patients’ tumor tissues but also to increase its clinical efficacy and minimize its systemic side effects [24]. For instance, adenovirus-delivered IL-12 gene in a cancer cell-restricted manner without overlapping toxicities has been demonstrated in a number of animal studies; however, the majority of these studies have also highlighted the importance of its combination with additional anticancer gene or therapeutic modality to further improve its overall anticancer properties [22–26].

Of note, the differences of their anti-cancer mechanisms can strongly support the potential benefit of TRAIL and IL-12-based combination therapy. In agreement, co-therapy with recombinant TRAIL and IL-12 proteins has been found to significantly sensitize HCC cells to TRAIL’s apoptotic effect [27]; and treatment with IL-12 has shown to upregulate TRAIL expression on NK cells and contributes to IFN-γ-dependent NK cell protection from tumor metastasis [28]. Based on these encouraging data, it therefore may be hypothesizing that their combined therapy through the strategy of cancer targeting dual gene virotherapy may renew interest and represent a meaningful therapeutic maneuver in cancer therapy. However, to best of our knowledge the reliability of such strategy for treatment of HCC has not been sufficiently investigated far. Therefore, in the present study we generated two OAds armed with human TRAIL and IL-12 gene (Ad-ΔB/TRAIL and Ad-ΔB/IL-12, respectively) and their combination therapy was assessed both in vitro on human HCC cell lines and in vivo on an orthotopic human HCC model induced in the liver lobules of nude mice. Overall, our results showed that combined therapy with Ad-ΔB/TRAIL plus Ad-ΔB/IL-12 had enhanced anti-HCC effect at the in vitro and in vivo levels, and was closely associated with enhanced activation of apoptosis and anti-tumor immunity and repression of tumor angiogenesis and vascularization.

## Methods

### Cell lines and culture conditions

The Hep3B human HCC cell line, the WRL68 human normal liver cell line, and the HEK293 human embryonic kidney cell line expressing the E1A region of Ad5 were obtained from the American Type Culture Collection (ATCC, Manassas, VA, USA), while the HuH7 human HCC cell line was obtained from Japan Health Science Research Resources (JCRB Genebank, Osaka, Japan). All cell lines were cultured in Dulbecco’s modified Eagle’s medium (DMEM; Thermo Fisher Scientific, Inc., Waltham, MA, USA) supplemented with 10 % fetal bovine serum (Gibco-BRL, Grand Island, NY, USA), 2 mmol/L glutamine, 50 units/ml penicillin, and 50 μg/ml streptomycin (Gibco-BRL, Grand Island, NY, USA). All cells were maintained at 37 °C in a humidified incubator with 5 % CO_2_.

### Generation and purification of oncolytic adenoviruses expressing human TRAIL or human ING4 transgene

A conditionally replication-competent oncolytic adenovirus (Ad-ΔB) mutated in E1A and deleted in E1B regions was generated as previously described [22]. To generate Ad-ΔB-expressing human TRAIL gene (Ad-ΔB/TRAIL) or human IL-12 gene (Ad-ΔB/IL-12), a DNA region of human TRAIL or IL-12 was first amplified by PCR with the following primer sets: hTRAIL; 5′-ATCGCCCGGATTAAGAAA-3′ (sense primer), 5′-CAAGTGCAAGTTGCTCAGGA-3′ (antisense primer), IL-12; 5′- CCTCCTTGTGGCTACCCTGG-3′ (hp35 sense primer), 5′- GAAGCATTCAGATAGCTCATCAC-3′ (hp35 antisense primer), 5′- AGCAAGATGTGTCACCA-3′ (hp40 sense primer), 5′-TTAGGTTCTGATCCAGGA-3′ (hp40 antisense primer). Hp35 and hp40 are the light and heavy chains of human IL-12, respectively. Each amplified PCR product was then sub-cloned into pSP72 Ad shuttle vector containing the E3 region of Ad type 5 (pSP72-E3; promega, Madison, WI) to generate a pSP72-TRAIL and a pSP72-IL-12 Ad shuttle vector, respectively. The shuttle vectors were then linearized with restriction enzyme digestion, co-transformed with Ad-ΔB DNA into *Escherichia coli* BJ5183, and cultured overnight for homologous recombination. To verify the respective homologous recombinants, the plasmid DNA purified from overnight *E. coli* culture was digested with *Hind*III, and the digestion pattern was confirmed by PCR analysis. The resultant homologous plasmid DNA recombinants were further digested with *Pac*I and transfected into HEK293 cells to generate Ad-ΔB/TRAIL and Ad-ΔB/IL-12. The three generated OAds (Ad-ΔB, Ad-ΔB/TRAIL, and Ad-ΔB/IL-12) were further propagated in HEK293 cells, purified by CsCl gradient density purification method, and then dissolved in storage buffer (10 mM Tris, 4 % sucrose, 2 mM MgCl_2_) and stored at−80 °C until use [29, 30]. The number of viral particle (Vp) for each virus was calculated from measurements of optical density at 260 nm (OD_260_), where one absorbency unit is equivalent to 1.1 × 10^12^ viral particles per milliliter (VP/mL) [30]. Infectious titers (plaque-forming units (PFU) per milliliter) were also determined by limiting dilution assay on HEK293 cells; the multiplicity of infection (MOI) was calculated from the infectious titers.

### Validation of TRAIL and IL-12 expression and viral replication in the transfected HCC cells

HuH7 human HCC cells were plated onto 24-well plates at a density of 4 × 10^4^ cells per well. After 24 h, the cells were treated with PBS, Ad-ΔB, or Ad-ΔB/TRAIL+ Ad-ΔB/IL-12 at a MOI of 5 for each virus in serum-free DMEM. After 48 h post-treatment, the cell lysates and conditioned medium were harvested and used to measure the expression pattern of TRAIL and IL-12 at their mRNA and protein levels by using Q-RT-PCR and ELISA assay, respectively. For Q-RT-PCR, total cellular RNA was extracted from each sample with TRIzol® reagent (Gibco BRL, Grand Island, NY) according to the manufacturer׳s instructions, and cDNA was prepared from 1 μg total RNA by random priming using a first-strand cDNA synthesis kit (Promega Corp., Madison, WI), under the following conditions: 95 °C for 5 min, 37 °C for 2 h, and 75 °C for 15 min. Q-RT-PCR reaction mixtures were assembled using the AmpiGene™ qPCR Green Mix Lo-ROX (Enzo Life Sciences, Switzerland), 10 pmol of primers and 50 ng of cDNA. The relative mRNA expression levels of TRAIL and IL-12 were determined with the following primer set: TRAIL; 5′- ATCGCCCGGATTAAGAAACT-3′ (sense primer), 5′- CAAGTGCAAGTTGCTCAGGA-3′ (antisense primer); IL-12; 5′- AAG GAG GCG AGG TTC TAA GC-3′ (hp40 sense primer), 5′- AAGAGCCTCTGCTGCTTTTG-3′ (hp40 antisense primer). Q-RT-PCR reactions were performed and analyzed using the ABI 7500 Real-Time PCR system (Applied Biosystems, Foster city, CA), and 18S ribosomal RNA was used as the endogenous control.

For ELISA, the samples were lysed in ice-cold RIPA buffer with a proteinase inhibitor cocktail (Sigma), centrifuged, and the concentrations of TRAIL and IL-12 proteins were individually measured in all supernatants by using conventional ELISA kits (BD, San Jose, CA) and following the manufacturers’ instructions.

These lysates of the treated HuH7 cells were also used for determination of viral production by measuring the expression of Ad E1A protein (as an indicator of viral replication) by western blotting using rabbit anti-Ad E1A primary antibody (Santa Cruz Biotechnology, Santa Cruz, CA) and as described previously [31].

### In vitro cell viability (MTT) assay

MTT [3-(4, 5-dimethylthiazol-2-yl)-2, 5-diphenyltetrazolium bromide; Sigma Chemical Co., St. Louis, MO, USA] assay was used to determine in vitro killing effect of Ad-ΔB/TRAIL+Ad-ΔB/IL-12 on two human HCC cell lines (Hep3B and HuH7) and compared with their effect on the normal human liver cells (WRL68) as previously described [29]. Briefly, the Hep3B, HuH7 and WRL68 cells were plated onto 24-well plates at about 60 to 70 % confluence, and then treated with PBS, Ad-ΔB, or Ad-ΔB/TRAIL+Ad-ΔB/IL-12 at the indicated MOIs (5 and 10 for Hep3B; 10 and 20 for Huh7; and 20 and 50 MOIs for WRL68). Cells were incubated at 37 °C for 48 h, and 200 μl MTT (2 mg/ml in PBS) was added to each well. After incubation for four hours at 37 °C with 5 % CO_2_, the supernatant and culture medium was aspirated and 150 μl Dimetylsulfoxide (DMSO) per well was added to dissolve the insoluble formazan crystals into a colored solution. Thereafter, the average spectrophotometric readings of the absorbance at 540 nm from three replicates of each treatment were determined in a microplate reader (Bio-Rad 680, Hercules, CA). Cell viability in each well was calculated according the following formula: cell survival = (absorbance value of treated cells-blank)/(absorbance value of untreated control cells), and expressed as the percentage of untreated control cells [29].

### In vitro cytopathic effect assay

To further confirm the cancer cell-cytopathic effects of the generated OAds, the human Hep3B and WRL68 cells (4 × 10^4^ cells per well) were cultured and grown to 60–70 % confluence in 24-well plates, and then treated with Ad-ΔB, or Ad-ΔB/TRAIL+ Ad-ΔB/IL-12 at various MOIs (1, 2, 5, 10, 20 in Hep3B cells; and 5, 10, 20, 50, 100 in WRL68), followed by incubation for 3 days at 37 °C allowing viral replication and cell-lysis. The media were replaced with 500 μl crystal violet solutions (0.5 % crystal violet in 50 % methanol) and 30 min later all wells were lightly washed with distilled water, naturally dried and documented as photographs. Each experiment was repeated three times, and PBS-treated cell groups were simultaneously analyzed and used as a negative control.

### In vitro analysis of apoptosis by flow cytometry

HuH7 cells (5 × 10^5^ cells per well) were cultured and treated with PBS, Ad-ΔB, or Ad-ΔB/TRAIL+Ad-ΔB/IL-12 (at MOI of 5) were incubated for 52 h. subsequently, the cells were washed once with complete medium, resuspended in binding buffer and then stained with Annexin V-fluorescein isothiocyanate (FITC; 0.5 μg/ml; as early apoptotic marker) and propidium iodide (0.6 μg/ml; as late apoptotic marker) (BioVision, Inc., Milpitas, CA), according to the manufacturer’s instructions. After 15 min incubation in the dark at room temperature, stained cells were immediately analyzed by flow cytometry (BD, San Jose, CA). All samples were assayed in triplicate, and total apoptotic cell population was calculated by the percentage of early apoptotic cells and late apoptotic cells.

### In vivo orthotopic mouse model of human HCC, treatment and experimental approaches

An orthotopic transplanted model of human HCC was established in 6- to 7 weeks-old male athymic nude mice as described previously [32, 33]. The model was induced by intrahepatic implantation of 2 × 10^6^ human Hep3B/fluc cells into the left liver lobes of mice, and the animals were maintained in laminar flow cabinets under pathogen-free condition, and all experimental protocols and procedures were in accordance with the EU Directive 2010/63/EU for animal experiments. Fifteen days post-Hep3B implantation, bioluminescence imaging was taken to confirm model establishment; in which mice were anesthetized in a chamber filled with 2 % isofluorane in oxygen and injected intraperitoneally with 150 mg/Kg D-luciferin potassium salt (Caliper, Hopkinton, MA), and then photographic and bioluminescent images were obtained from the anesthetized mice using IVIS imaging system (Xenogen, Alameda, CA). In vivo bioluminescence signals were calculated as the sum of both prone and supine acquisitions for each mouse after background subtraction total flux (photons/s) from a total body ROI (region of interest) as described previously [32]. Survival mice that their imaged signals reached around 2 × 10^8^ p/s were enrolled as positive HCC-bearing mice, and were thereafter randomized into three groups (*n* = 10/group) and systemically treated with: PBS alone (Group 1), Ad-ΔB (Group 2), and Ad-ΔB/TRAIL + Ad-ΔB/IL-12 (Group 3). The dosage regimen of each OAd was 1 × 10^10^ VP (diluted in 200 μL PBS) injected intravenously through the caudal vein and repeated three times every other day [29]. Three days after administration of the last dose of each treatment strategy, four mice from each group were randomly selected, euthanized under general anesthesia (sodium pentobarbital; 120 mg/kg, i.p.), and their blood samples and tumor tissues were harvested and employed for the following purposes: (1) validation of intratumor viral replication (represented by viral E1A protein expression) and expression of TRAIL and IL-12 transgenes at their mRNA and protein levels by the same methodology as described above; (2) histopathological, immunohistochemical and *in situ* apoptosis analyses; (3) measuring of intratumor expression patterns of IFN-γ and VEGF, caspase-3 and 8; and (4) measuring of serum levels of hepato-renal function biomarkers. Of note, the remaining mice (*n* = 6 per group) were monitored weekly by bioluminescence imaging to determine the pattern of tumor response to the subjected treatments until the end of study (day 14 post-the last treatment dose).

### Histopathology, immunohistochemistry and TUNEL assay

Specimens of the harvested livers and tumor tissues were fixed in 4 % formalin followed by embedding in paraffin. Tissue sections (4 μm) were subjected to histopathology (H&E staining), immunohistochemical (IHC) staining and *in situ* apoptosis detection by TUNEL assay. For IHC, after dewaxing, antigen retrieval and endogenous peroxidase blocking steps the sections were subjected to staining procedures with purified monoclonal anti-mouse CD11b (BD PharMingen, San Jose, CA); anti-mouse NK1.1 (Biolegend, San Diego, CA); and anti-rabbit CD31 (Abcam, Cambrige, MA) primary antibodies, respectively. Subsequently, biotinylated goat anti-mouse or anti-rabbit immunoglobulins as secondary antibodies and streptavidin peroxidase complex reagent were applied. The visualization signals were developed with diaminobenzidine (DAB) chromogen substrate (DAKO, Carpinteria, CA and the slides were counterstained with Meyer’s hematoxylin and dehydrated through a series of ethanol and xylenes. In addition, intratumor microvessel density was microscopically counted in absolute values as previously described [29, 34]. Finally, for detection of *in situ* apoptosis in the tumor tissues, TUNEL (terminal deoxynucleotidyl transferase (TdT)-mediated dUTP nick-end labeling) assay was performed according to the manufacturer’s protocol (Roche Molecular Biochemicals) as described previously [34].

### Western blot assay of caspases-3 and-8 in the tumor tissues

Protein expression of two common apoptosis-related molecules, caspase-3 and caspase—8, was determined in the harvested tumor tissues treated with PBS, with Ad-ΔB, or Ad-ΔB/TRAIL+ Ad-ΔB/IL-12 by using western blot analysis to identify a possible underlying mechanism involved in treatment-mediated apoptosis. Briefly, each sample was lysed in ice-cold RIPA buffer with a proteinase inhibitor cocktail, centrifuged in a high-speed microcentrifuge for ten minutes, and then the total protein content was determined in the extract using a BCA protein assay reagent kit (Pierce, Rockford, IL). For each protein sample, 20 μg was separated by 10 % sodium dodecyl sulfate–polyacrylamide gel electrophoresis and transferred onto a PVDF membrane (Millipore). The membranes were blocked in TBST (50 mM Tris–HCl, pH 7.6, 150 mM NaCl, 0.2 % Tween 20) containing 3 % BSA for 2 h, and then incubated with rabbit polyclonal IgG primary antibodies to caspase-3 and caspase-8 (Cell signaling, Danvers, MA), and β-actin (Santa Cruz Biotechnology, Santa Cruz, CA), for overnight at 4 °C. Subsequent incubation with goat anti–rabbit IgG- horseradish peroxidase (HRP) secondary antibodies was performed for 45 min at room temperature, followed by 3–5 washes with TBST buffer, and then the blots were developed with the enhanced chemiluminescence detection reagents.

### ELISA assays of IFN-γ and VEGF in the tumor tissues

Liver tumor tissue specimens of HCC-bearing mice treated with PBS, Ad-ΔB, or Ad-ΔB/TRAIL + Ad-ΔB/IL-12 were homogenized in ice-cold RIPA lysis buffer with a proteinase inhibitor cocktail (Santa-Cruz Biotechnology Inc, Burlingame, CA) and analyzed for total protein content using a BCA protein assay reagent kit (Pierce). Concentrations of IFN-γ and VEGF in the tumor tissue lysates were quantitatively measured by using commercially available ELISA kits (R&D systems, Minneapolis, MN, USA) and according to manufacturer’s instructions.

### In vivo safety study

At 72 h after the last injected dose of each therapeutic strategy, blood samples were collected and their obtained sera were prepared form their representatives whole blood samples and biochemically analyzed to measure the serum levels of liver function parameters (ALT AST, and bilirubin), and kidney function parameters (BUN and creatinine) according to their standard protocols. In addition, the harvested liver tissues of the different animal groups were subjected to H&E staining and microscopic examination to assess the possible changes in their liver morphology.

### Statistical analysis

Data were expressed as mean ± SEM. Statistical comparison was analyzed by one-way analysis of variance (*ANOVA*) using the Statistical Package for the Social Sciences (SPSS) program, version 18, followed by *Mann–Whitney* test to compare significance between groups. The difference between data were considered to be statistically significant when *P* <0.05; very significant when *p* <0.01; and very much significant when *p* <0.001.

## Results

### In vitro co-therapy with Ad-ΔB/TRAIL plus Ad-ΔB/IL-12 exhibited efficient viral replication and TRAIL and IL-12 expression in human HCC cells

**Fig. 1:**
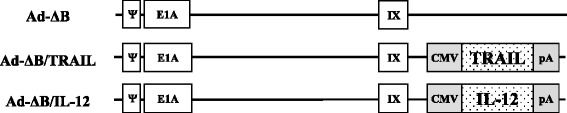
Graphical representation of the constructed and generated non-armed control oncolytic adenovirus (Ad-ΔB) and oncolytic adenovirus armed with human TRAIL gene (Ad-ΔB/TRAIL) or human IL-12 gene (Ad-ΔB/IL-12). TRAIL or IL-12 gene was incorporated in the E3 region of the viral backbone under the transcriptional control of the human cytomegalovirus (CMV) promoter

**Fig. 2:**
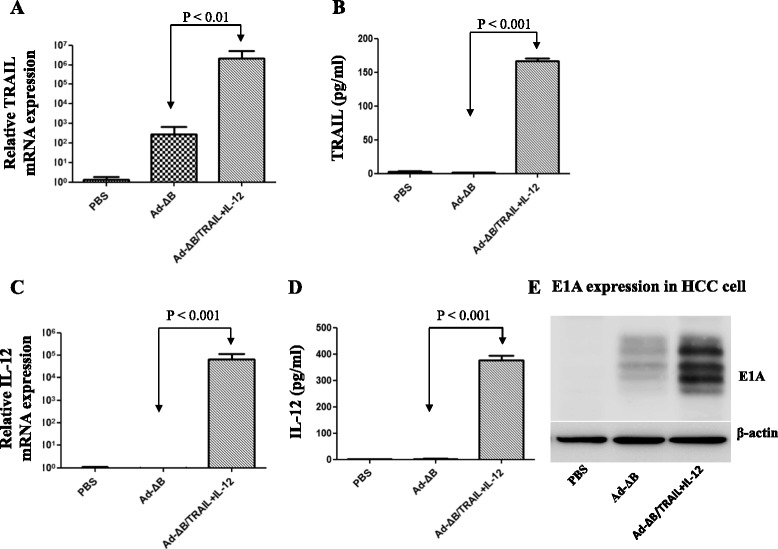
Validation of in vitro oncolytic adenoviral replication efficiency and the expression patterns of the encoded TRAIL and IL-12 transgenes in human HCC cells. HuH7 cells were cultured and then treated with PBS, Ad-ΔB, or Ad-ΔB/TRAIL+ Ad-ΔB/IL-12 at a MOI of 5 for each virus. Two days post-treatment, the cell lysates and conditioned media were harvested and relative mRNA and protein levels of TRAIL (**a** and **b**), or IL-12 (**c** and **d**) were measured by using Q-RT-PCR and ELISA assays, respectively. Data are represented as mean ± SE. In addition, viral production was determined by measuring the expression of Ad E1A protein (**e**) by western blot assay using rabbit anti-Ad E1A primary antibody

In the present study, a control non-armed OAd (Ad-ΔB) and two independent armed OAds expressing human TRAIL (Ad-ΔB/TRAIL) and human IL-12 (Ad-ΔB/IL-12) transgene were generated (Fig. 1). OAds replication efficiency and the expression pattern of TRAIL and IL-12 transgenes were measured in the human HuH7 HCC cells treated with PBS, Ad-ΔB, or Ad-ΔB/TRAIL+ Ad-ΔB/IL-12 at a MOI of 5 for each Ad. Both mRNA and protein levels of either TRAIL or IL-12 was significantly detected only in HuH7 cells co-transfected with Ad-ΔB/TRAIL plus Ad-ΔB/IL-12 (Fig. 2a-d). Similarly, there was remarkable expression of viral E1A protein (as an indicator of viral replication) in HCC cells treated with Ad-ΔB/TRAIL+ Ad-ΔB/IL-12 combination therapy than those treated with PBS-or Ad-ΔB (Fig. 2e). This in turn meant that co-treatment with Ad-ΔB/TRAIL and Ad-ΔB/IL-12 exhibited simultaneous and efficient expression of both TRAIL and IL-12, and also didn’t interfere with viral replication, in human HCC cells.

### In vitro co-therapy with Ad-ΔB/TRAIL plus Ad-ΔB/IL-12 exhibited profound anti-proliferative and cytopathic effects on human HCC cells

**Fig. 3:**
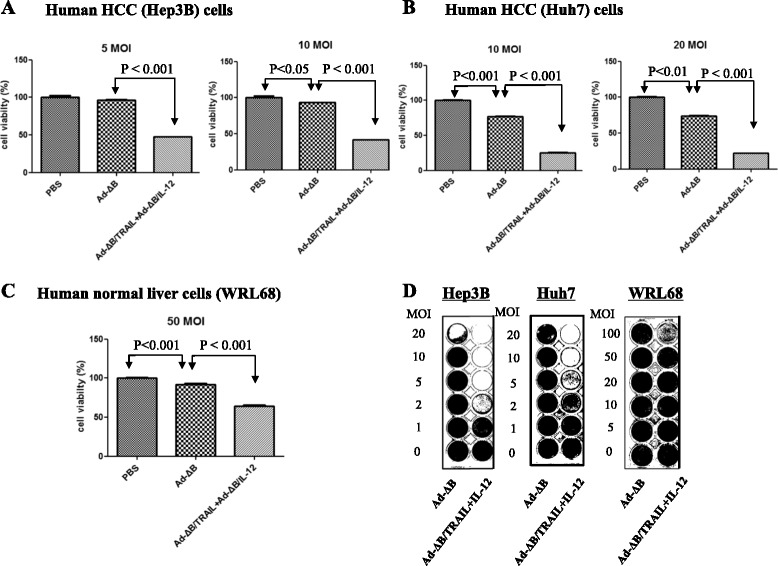
In vitro effects of Ad-ΔB and Ad-ΔB/TRAIL+Ad-ΔB/IL-12 on Hep3B and HuH7 human HCC cells and normal human liver (WRL68) cells. **a** Quantitative results of MTT assay showing the inhibitor effects on viability of Hep3B cells at 5 and 10 MOI (the multiplicity of infection) per virus; (**b**) Quantitative results of MTT assay showing the inhibitor effects on viability of HuH7 cells at 10 and 20 MOI per virus; and (**c**) Quantitative results of MTT assay showing the inhibitor effects on viability of WRL68 cells at 50 MOI per virus. Data are represented as mean ± SE. **d** Representatives of semi-quantitative assessment of cytotoxic potency by crystal violet cytopathic effect assay on Hep3B cells (left panel), HuH7 cells (middle panel), and WRL68 cells (right panel) at indicated MOIs. Ad-ΔB = non-armed control oncolytic adenovirus; Ad-ΔB/TRAIL+Ad-ΔB/IL-12 (or Ad-ΔB/TRAIL+IL-12) = combined therapy with two oncolytic adenoviruses encoding human TRAIL and IL-12 genes, respectively

We next measured the in vitro suppressive effects of Ad-ΔB and Ad-ΔB/TRAIL+Ad-ΔB/IL-12 on the proliferation and viability of two human HCC cell lines (Hep3B & HuH7) by using MTT and crystal violet assays. Results of MTT assay demonstrated that in a dose-dependent manner the combined treatment with Ad-ΔB/TRAIL+Ad-ΔB/IL-12 had resulted in potent killing effects on the tested Hep3B (Fig. 3a) and HuH7 (Fig. 3b) human HCC cell lines than those achieved by tretament with Ad-ΔB. The same findings were also confirmed with crystal violet assay (Fig. 3d), whereby co-treatment with Ad-ΔB/TRAIL and Ad-ΔB/IL-12 elicited more cytopathic effect on HCC cells than Ad-ΔB. Furthermore, to evaluate their in vitro safety, the normal human liver cell lines (WRL68) were infected with Ad-ΔB or Ad-ΔB/TRAIL+Ad-ΔB/IL-12 at doses of 20 and 50 MOIs in MTT assay (Fig. 3c), and from 1 to 100 MOIs in crystal violet assay (Fig. 3d), and the collective results showed that Ad-ΔB/TRAIL+Ad-ΔB/IL-12 showed potent cancer cell killing efficacy in HCC cells (Hep3B and Huh7), which was markedly higher to that of Ad-ΔB. In contrast, no high significant difference between the cytopathic effects of Ad-ΔB and Ad-ΔB/TRAIL+Ad-ΔB/IL-12 on WRL68 cells at their doses used to induce killing effect on HCC cells. Together, these results indicate that dual treatment with Ad-ΔB/TRAIL plus Ad-ΔB/IL-12 can induce potent and HCC-selective cancer cell killing effect with minimal cytotoxicity in normal liver cells.

### Potent in vivo anti-tumor effect of Ad-ΔB/TRAIL/Ad-ΔB/IL-12 combination therapy

**Fig. 4:**
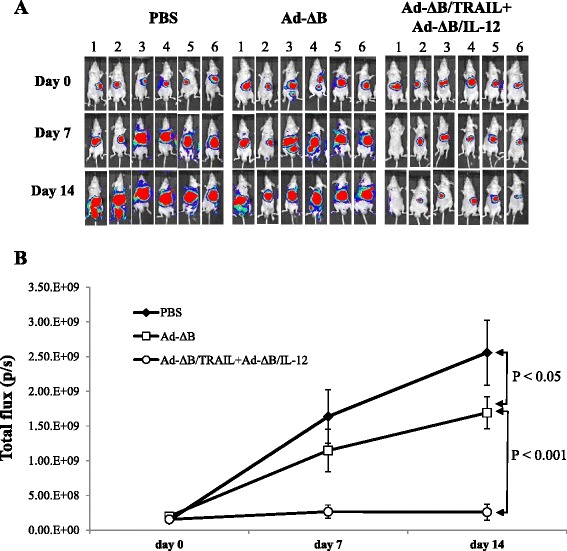
In vivo antitumor effects of Ad-ΔB and Ad-ΔB/TRAIL+Ad-ΔB/IL-12 on an orthotopic transplanted mouse model of human HCC. The model was induced by intrahepatic implantation of 2 × 10^6^ human Hep3B/fluc cells into the left liver lobes of mice in athymic nude mice. Positive HCC-bearing mice were thereafter randomized into three groups (*n* = 10/group) and through their tail veins they were treated with: PBS alone (Group 1), Ad-ΔB (Group 2), and Ad-ΔB/TRAIL+Ad-ΔB/IL-12 (Group 3) at a dosage regimen of 1 × 10^10^ VP in 200 μL PBS of each virus; repeated three times every other day. Next, the mice were monitored and photographed (**a**) weekly by in vivo bioluminescence imaging to determine the pattern of tumor response to the subjected treatments (**b**) until the end of study (day 14 post-the last treatment dose)

**Fig. 5:**
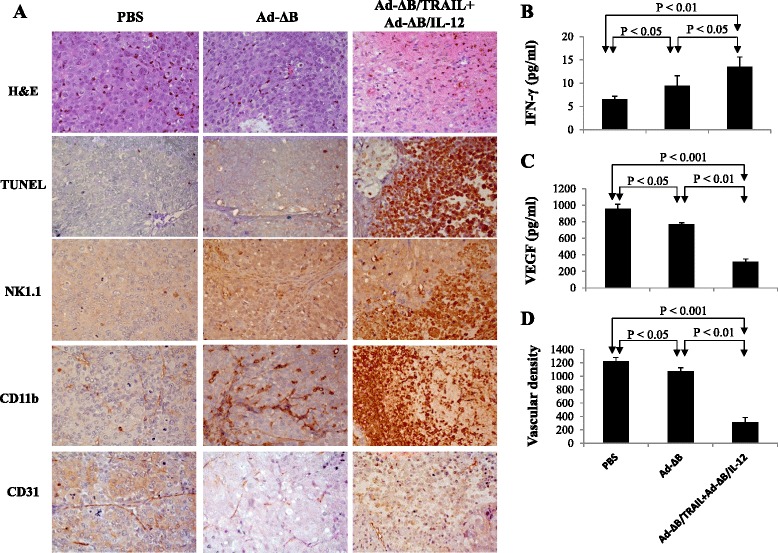
Histopathological, immunohistochemical and apoptosis assessments of the harvested tumor tissues from different animal groups. Three days post-treatment of HCC-bearing mice with PBS, Ad-ΔB, or Ad-ΔB/TRAIL+Ad-ΔB/IL-12 (at a dosage regimen of 1 × 10^10^ VP in 200 μL PBS of each virus; repeated three times every other day), the mice were euthanized under general anesthesia and their liver tumor tissues were harvested and prepared for histopathological, immunohistochemical (IHC) and apoptosis assessments, and measurement of intra-tumor levels of IFN-γ and VEGF. **a** Photographs of H&E: hematoxylin and eosin staining of tumor tissues for histopathology; TUNEL: Terminal uridine deoxynucleotidyl transferase dUTP nick end labeling staining assay of apoptotic cells in tumor tissue; NK1.1: IHC staining of infiltrated natural killer cells; CD11b: IHC staining of recruited antigen-presenting cells (dendritic cells and macrophage); and CD31: IHC staining of tumor CD31-positive microvessels endothelial cells. Original magnification: × 400. b and c are quantitative ELISA assays of the intratumor expression levels of IFN-γ and VEGF, respectively, at their protein levels. **d** The mean microvessel density for each treatment group was determined by counting CD31-positive vessels in 10 high-power fields. Each experiment was performed at least three times, and data shown are from representative experiments. Values of (**b**), (**c**) and (**d**) represent the mean ± SE

An in vivo orthotopic mouse model of human HCC was used to demonstrate the anti-tumor effect of the different treatments. Two weeks after intrahepatic implantation of 2 × 10^6^ human Hep3B/fluc cells, bioluminescence signal was done to confirm model establishment. Mice that their imaged signals reached around 2 × 10^8^ p/s were thereafter enrolled as positive HCC-bearing mice and then randomized into three groups and intravenously treated with: PBS, Ad-ΔB, and Ad-ΔB/TRAIL+Ad-ΔB/IL-12, respectively, at a dosage schedule of 1 × 10^10^ VP of each vector/injection; three times with two days interval. In vivo tumor bioluminescence imaging were subsequently taken on days 0, 7, day 14 post-the last injected dose of each treatment to determine the tumor response for each treatment. As shown in Fig. 4, combined treatment with Ad-ΔB/TRAIL plus Ad-ΔB/IL-12 showed marked inhibitory effect on the intrahepatic implanted tumors; and after 14 days, this combination therapy had resulted in 6.5-fold greater reduction in the tumor bioluminescence imaging (*P* <0.001) compared to a therapy with Ad-ΔB (Fig. 4). Findings of the histopathological (H&E) examination of the tumor tissues that were collected 3 days after the last injected dose of each treatment were in full harmony with in vivo bioluminescence imaging results and revealed the presence of a large area of proliferating tumor cells without necrotic changes in the tumor tissues of mice treated with PBS (Fig. 5a). By contrast, large necrotic areas were remarkably observed in the tumor tissues of mice co-treated with Ad-ΔB/TRAIL plus Ad-ΔB/IL-12; reflecting their extensive in vivo tumouricidal effect. However, HCC-bearing mice treated with Ad-ΔB showed only a few necrotic cells in their tumor tissues (Fig. 5a).

### Combined therapy with Ad-ΔB/TRAIL and Ad-ΔB/IL-12 is associated with induction of antitumour immune response and inhibition of tumour angiogenesis

To understand the possible underlying mechanisms behind the efficient anti-HCC effect of Ad-ΔB/TRAIL+Ad-ΔB/IL-12 combination therapy, the expression level of three cellular markers; NK1.1-, CD11b-, CD31- positive cells, and the change in the intratumor microvascular density was examined by IHC. Additionally, the expression levels of IFN-γ and VEGF were also measured in the tumor tissues by ELISA assay. As demonstrated in Fig. 5a, cellular markers of antitumor immune cells such as natural killer cells (NK1.1-positive cells) and antigen presenting cells (monocyte/macrophages and dendritic cells; CD11b-positive cells) were abundantly infiltrated into tumor tissues of mice treated with Ad-ΔB/TRAIL plus Ad-ΔB/IL-12 compared with therapy with Ad-ΔB (Fig. 5a). These IHC observations were also associated with significant upregulation of IFN-γ production (Fig. 5b) and downregulation of VEGF expression (Fig. 5c) in the harvested HCC tumor tissues. Constantly, a marked decrease in blood vessels expressing CD31-positive cells (Fig. 5a) and a significant suppression of vascular density (Fig. 5d) was also observed in the tumor tissues of animals received Ad-ΔB/TRAIL+Ad-ΔB/IL-12 combination therapy than other treated groups. In short, these findings suggest that concomitant therapy with Ad-ΔB/TRAIL plus Ad-ΔB/IL-12 was significantly associated with generation of anti-tumour specific immune response as well as anti-angiogenesis and antitumor vasculature effect than therapy with Ad-ΔB.

### Ad-ΔB/TRAIL and Ad-ΔB/IL-12 exhibited efficient viral replication and expression of their armed TRAIL and IL-12 transgenes in the tumor tissues

**Fig. 6:**
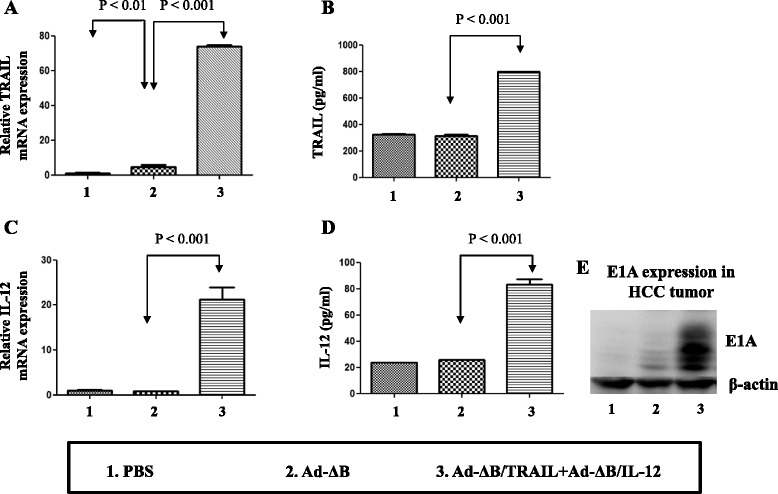
Validation of intra-tumor oncolytic adenoviral replication and the expression patterns of the encoded TRAIL and IL-12 transgenes. Three days post-treatment of HCC-bearing mice with PBS, Ad-ΔB, or Ad-ΔB/TRAIL+Ad-ΔB/IL-12 (at a dosage regimen of 1 × 10^10^ VP in 200 μL PBS of each virus; repeated three times every other day), the mice were euthanized under general anesthesia and their liver tumor tissues were harvested and investigated to validate intratumor viral replication and expression of TRAIL and IL-12 transgenes. **a** and **b** represents intra-tumor TRAIL expression at its mRNA and protein level, respectively, and (**c**) and (**d**) represents intra-tumor IL-12 expression at its mRNA and protein level, respectively. Data are represented as Mean ± SE. **e** represents the expression of Ad E1A protein (an indicator of viral production) by western blot assay

Furthermore, molecular analysis (Fig. 6a-e) showed that tumor tissues of animals treated with Ad-ΔB/TRAIL+Ad-ΔB/IL-12 combined therapy elicited profound elevation in the mRNA and protein levels of TRAIL and IL-12 transgenes (Fig. 6a-d), as well as in the degree of intratumor viral replication as reflected by the expression level of E1A protein (Fig. 6e); suggesting that the strategy of OAd-mediated TRAIL and IL-12 dual gene transfer exhibited simultaneous and efficient expression of the encoded transgenes and didn’t interfere with viral replication in human HCC tumor tissues.

### Combined therapy with Ad-ΔB/TRAIL and Ad-ΔB/IL-12 induces apoptosis and caspases-3 and 8 in both HCC cells and tumors

**Fig. 7:**
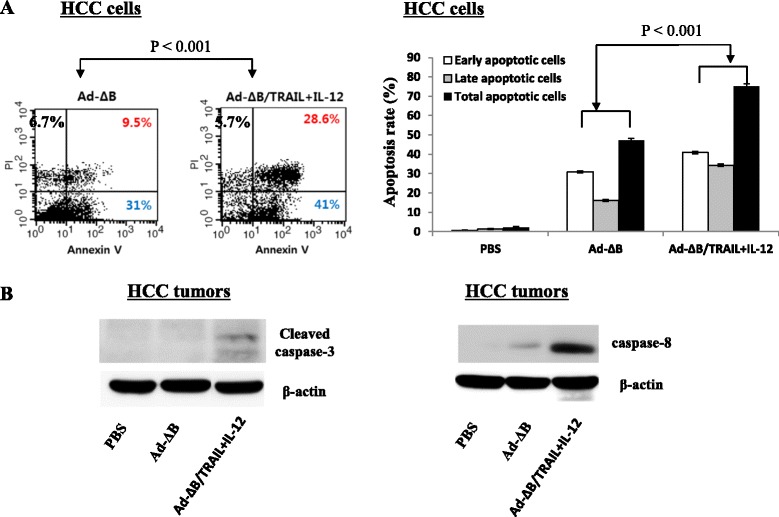
In vitro and in vivo apoptotic findings. **a** Flow cytometric analysis of apoptosis induction in HCC cells-treated Ad-ΔB/TRAIL plus Ad-ΔB/IL-12 (Ad-ΔB/TRAIL+IL-12), in comparison with cells treated with control Ad-ΔB or PBS, by using Annexin-V and propidium iodide (PI) fluorescence staining assay. Each scatter plot demonstrates the percentage of early apoptotic cells (Annexin-V^+^cells, bottom right quadrant) and late apoptotic cells (PI^+^cells, upper right quadrant). **b** Western blot analysis showing the intra-tumor expression levels of cleaved caspase-3 (left panel) and caspase-8 (right panel) at day 3 after treatment of HCC-bearing mice with PBS, Ad-ΔB, or Ad-ΔB/TRAIL + Ad-ΔB/IL-12 (Ad-ΔB/TRAIL+IL-12) at a dosage regimen of 1 × 10^10^ VP in 200 μL PBS of each virus; repeated three times every other day

To further explore the mechanisms that might be involved in the anti-HCC therapeutic effects of the two tested treatment approaches, we examined the apoptotic changes both in vitro on HuH7 cells and in vivo on the tumor tissues that were collected 3 days after the last treatment dose with either Ad-ΔB or Ad-ΔB/TRAIL + Ad-ΔB/IL-12 combination therapy. As demonstrated in Fig. 7a, in vitro co- treatment with Ad-ΔB/TRAIL and Ad-ΔB/IL-12 had resulted in approximately 69.6 % of total Huh7 cell apoptosis (41 % early apoptotic cells and 28.6 % late apoptotic cells), compared to approximately 40.5 % of apoptotic Huh7 cells when treated with Ad-ΔB (31 % early apoptotic cells and 9.5 % late apoptotic cells). In agreement with these in vitro results, TUNEL assay, that was applied to detect *in situ* apoptotic cells in the tumor tissues, revealed that the combination of Ad-ΔB/TRAIL plus Ad-ΔB/IL-12 had resulted in a more significant induction of intratumor apoptosis compared to therapy with Ad-ΔB (Fig. 5a). This meant that oncolytic Ad with TRAIL and IL-12 genes were interacted together to eradicate human HCC cells and tumors by, at least in part, induction of apoptosis phenomenon. Next, we proceeded to address the possible underlying molecular mechanism that could be involved in apoptosis promotion. Towards this goal, the activity caspase-3 and caspase-8, well-known apoptosis-inducing molecules, were measured in the harvested tumor tissues by western blotting analysis. Our findings demonstrate that tumors treated with a combination of Ad-ΔB/TRAIL and Ad-ΔB/IL-12 had significant elevations in cleaved caspase-3 and caspase-8, than those treated with the non-armed control Ad-ΔB (Fig. 7b). These results may therefore imply that the observed apoptotic effect was, partly, mediated by activating caspase cascade in HCC cells and tumour tissues.

### Biochemical and histological findings of in vivo safety study

At 72 h after the last injected dose of each treatment, the serum levels of liver and kidney function parameters were assessed. Interestingly, as shown in Table 1, there were no significant differences between the hepato-renal effects of the administered PBS, Ad-ΔB, nor Ad-ΔB/TRAIL+Ad-ΔB/IL-12; with one interest exception that therapy with Ad-ΔB/TRAIL+Ad-ΔB/IL-12 showed more safety on AST levels than therapy with non-armed Ad-ΔB. In addition, we examined the morphological changes in the liver tissues of HCC-bearing mice following systemic treatment with PBS, Ad-ΔB, or Ad-ΔB/TRAIL + Ad-ΔB/IL-12 (1 × 10^10^ VP, three times every other day) for further assessment of Ad-induced hepatotoxicity. As shown in Additional file 1: Figure S1, the livers harvested from oncolytic Ad-treated mice (Ad-ΔB or Ad-ΔB/TRAIL + Ad-ΔB/IL-12) shared similar morphology as the PBS-treated group, indicating that oncolytic Ads did not induce observable hepatotoxicity. Together, these results indicate that combination treatment with Ad-ΔB/TRAIL + Ad-ΔB/IL-12 did not induce observable hepatotoxicity, suggesting it as a safe and effective platform for the treatment of hepatocellular carcinoma.

**Table 1:** Biochemical findings

Treatment	Liver function biomarkers			Kidney function biomarkers	
	AST (Unit/L)	ALT (Unit/L)	Bilirubin (mg/dl)	BUN (mg/dl)	Creatinine (mg/dl)
PBS	168 ± 63.3	67 ± 27.5	0.1	27.0 ± 1.7	0.21 ± 0.02
Ad-ΔB	190 ± 36.4	52 ± 17.8	0.1	29.3 ± 5.5	0.20 ± 0.01
Ad-ΔB/TRAIL + Ad-ΔB/IL-12	118 ± 12.5	52 ± 42.2	0.2	30.6. ± 2.1	0.21 ± 0.02

HCC-bearing mice were systemically treated with PBS, controlled non-armed Ad-ΔB, or combination of Ad-ΔB/TRAIL+Ad-ΔB/IL-12; at a dosage regimen of 1 × 10^10^ VP/intravenous injection; three times every other day. 72 h after the last dose, serum levels of hepato-renal function biomarkers of each group were assessed. Data are represented as mean ± SE

## Discussion

Hepatocellular carcinoma (HCC) is one of the most common deadliest cancers and its annual incidence continues to rise globally [1]. Currently, there are several limitations in—and resistance to the therapy of HCC. Thus, there is a necessary need for development of new and more effective therapeutic alternatives [2–4]. To date, cancer virotherapy, particularly mediated by oncolytic viruses (OAds), have emerged as a novel effective strategy in the field of cancer therapy [6, 7, 11]. More importantly, their combination with other treatment options, in particular with cancer gene therapy, has been shown to strength antitumor efficacy through viral amplification of the therapeutic transgene inside the tumor cells and tissues [5–10]. With this concept, among several kinds of transgenes are being investigated, both TRAIL; as a unique antitumor apoptosis inducing agent [17–19], and IL-12; as a powerful antitumor immunostimulatory cytokine [23–26], seemed to represent the most promising candidates in the theme of cancer gene therapy. However, most of the previously published reports have elucidated that single therapeutic modality, including those mediated by TRAIL or IL-12 alone, could not pursue in the future to achieve sufficient antitumor responses and thus utilization of combination of anti-anticancer tools, such as those mediated by dual gene-based cancer therapy, holds the great promise for the future of cancer eradication [13, 14, 19, 23, 27, 35]. To the best of our knowledge the anti-cancer therapeutic potentiality of OAd virotherapy strategy-mediated co-delivery of TRAIL and IL-12 genes has not been sufficiently investigated far. Therefore, the present study was designed to evaluate the therapeutic effect of a combined therapy with two armed OAds (Ad-ΔB/TRAIL and Ad-ΔB/IL-12) against the human HCC at the preclinical level. Interestingly, our results showed that OAd-mediated simultaneous delivery of TRAIL and IL-12 genes elicited profound anti-HCC tumouricidal effects both in vitro and in vivo; and it was closely associated with enhanced apoptosis promotion, activation of anti-tumor immunity and inhibition of tumor angiogenesis and vasculature, than those mediated by non-armed control OAd lacking the therapeutic genes.

In the present study, the anti-HCC effect of combined therapy with Ad-ΔB/TRAIL+Ad-ΔB/IL-12 were measured both in vitro on human Hep3B and HuH7 cell lines; which are well differentiated human hepatocyte-derived cellular carcinoma cell lines commonly used in studying liver cancer and its potential therapies [32, 36], and in vivo on an orthotopic model of human HCC established by intrahepatic implantation of Hep3B into the liver lobes of immunodeficient nude mice. Data of our in vitro (MTT and crystal violet assays) and in vivo (bioluminescence imaging and H&E examination) studies disclosed that co-therapy with Ad-ΔB/TRAIL+Ad-ΔB/IL-12 resulted in not only cytopathic and anti-viability effects on HCC cells but also in marked eradication of the implanted tumor cells. In agreement with our findings, He et al. [27] have previously demonstrated that combination of recombinant TRAIL and IL-12 significantly sensitizes HCC cells to TRAIL’s apoptotic effect, and similarly Cai et al. [14] have elucidated the synergistic antitumor effect of TRAIL with another immunostimulant cytokine, IL-24, in eradication of subcutaneous xenograft model of hepatoma induced by BEL7404 cells. Likewise, Chiba et al. [35] suggested that IL-27, a member of IL-12 cytokine family, enhances the expression of TRAIL in human melanomas and inhibits their tumor growth partly in a TRAIL-dependent manner. Next, to clarify the efficacy of the administered OAds to replicate and to express their encoded TRAIL and IL-12 transgenes, we measured the expression levels of TRAIL and IL-12 proteins as well as the levels of adenoviral E1A protein (as an indicator of viral replication) in HCC cells and tumor tissues of different treated groups and the results indicated that co-treatment with Ad-ΔB/TRAIL and Ad-ΔB/IL-12 exhibited simultaneous and efficient expression of both TRAIL and IL-12 along with viral replication. This phenomenon has also been previously observed with other types of OAds co-expressing two antitumor genes [13]. Taken together, our findings can support the recently emerged hypothesis that dual or multi-gene-mediated oncolytic virotherapy, in which two independent therapeutic genes are simultaneously expressed by OAds, provide a more satisfactory anti-cancer therapeutic potential to target the cancer on several levels and pathways and to deal with the complexity of the tumor microenvironments through a triplex anticancer effect achieved by the viral oncolytic effect and the additive anti-cancer interactions between the encoded two transgenes [13–15].

Cancers that are detected clinically must have evaded antitumor immune responses to grow progressively, and therefore the prospect of effective immunotherapies for the treatment of patients with cancer is now becoming a clinical reality [37]. Towards this goal, and to address the underlying mechanisms whereby the combined therapy with Ad-ΔB/TRAIL and Ad-ΔB/IL-12 cooperatively inhibit the orthotopic model of HCC, we therefore investigated their effects on anti-tumor immune response represented by the degree of infiltrated natural killer cells (NK1.1 cells) and antigen presenting cells (dendritic cells, monocyte and macrophages; CD11b- cells), as well as by the concentrations of an antitumor-specific cytokine, IFN-γ, in the harvested tumor tissues at day 3 after the last treatment of HCC-bearing mice with either PBS, Ad-ΔB, or Ad-ΔB/TRAIL and Ad-ΔB/IL-12. Our findings demonstrated that both NK1.1-and CD11b-positive cells were abundantly infiltrated into tumor tissues of mice treated with Ad-ΔB/TRAIL plus Ad-ΔB/IL-12 compared with therapy with Ad-ΔB or PBS, and this effect was also accompanied with a remarkable upregulation in intratumoral IFN-γ production; reflecting that dual therapy with OAds co-expressing TRAIL andIL-12 is significantly associated with generation of anti-tumour specific immune response. In that respect, IL-12 has emerged as a potent inducer of antitumor immunity and was originally identified as NK cell stimulatory cytokine [23]; and IFN-γ is the central mediator of IL-12-induced antitumor activities [23]. Additionally, IL-12 has shown to upregulate TRAIL expression on NK cells [28] and enhance TRAIL-induced apoptosis in human HCC cells isolated from HCC-bearing patients [27]. Similarly, oncolytic virotherapy is a potent NK cell activator; and now there is strong evidence that NK cells are able to directly kill tumor cells not only through IFN-γ pathway but also through ligation of TRAIL receptors and other mechanisms [38, 39]. Blocking of TRAIL activity (.e.g., by neutralizing antibodies) has been found to significantly attenuate the cytotoxicity of human NK cells induced by IL-12; suggesting that the expression of TRAIL is one of the major mechanisms of NK cytotoxicity induced by IL-12 [40]. More importantly, increased number of NK cells in the liver tumor tissues of HCC patients is positively correlated with their survival and good prognosis and by contrast, dysfunction or exhaustion of NK cells to produce cytotoxicity and to secret IFN-γ in the tumor sites crucially contribute to the progression and invasion of HCC disease [41]. IFN-γ induces TRAIL expression in NK cells, and synergistic interactions between IFN-γ and TRAIL to eradicate tumors have also been previously reported [42]. Likewise, the antigen presenting cells, particularly dendritic cells (DCs), exhibit differential anti-tumorigenic functions and play a key role in inducing and maintaining the antitumor immunity [37]. Moreover, intratumoral delivery of a TRAIL-expressing OAd has been found to increase the production of IFN-γ and other cytokines acting as maturation signals for DCs as a therapeutic benefit of engaging DCs activity in cancer virotherapy [39]. IFN-γ acts on APCs to initiate or increase IL-12 secretion in a positive feedback loop [23], and delivery of IL-12 gene increases DCs proliferation and their intra-tumor infiltration through an IFN-γ-dependent pathway [22, 43] have also been reported. More interestingly, early reports have demonstrated that NK cells also enhance IFN-γ production by monocytes [38]; and like NK cells, there is a subset of DCs can produce IFN-γ and mediate TRAIL-dependent lysis of tumor cells [44]. Based on our findings and on these previous facts we can therefore suggest that there is a circadian crosstalk between OAds and TRAIL and IL-12 in induction of specific anti-tumor immunity.

Angiogenesis is central to the growth and metastasis of cancers; and VEGF is the key driver of tumor neovascularization, progression, and malignant phenotype [29, 45]. Additionally, cluster of differentiation 31 (CD31) also plays a complex role in tumor angiogenesis [46]. These observations provide a strong reason for the importance of VEGF and CD31 as a potential targets in modern cancer therapy by developing strategies that can inhibit VEGF and/or to disrupt its signaling pathway [45, 46]. Herein, our findings were in harmony and elucidated that Ad-ΔB/TRAIL and Ad-ΔB/IL-12 combination treatment was associated with a clear reduction in the intratumor expression of VEGF, CD31-positive cells, and in the microvessel density than the effects mediated by Ad-ΔB alone; suggesting a co-operative interaction between the administered OAds and their delivered TRAIL and IL-12 genes in halting tumor-driven angiogenesis and neovascularization. In agreement, earlier reports have demonstrated that OAds can attenuate tumor-driven angiogenesis [47]. Similarly, it has been demonstrated that TRAIL; via caspase-8-mediated functions [48], and IL-12; via IFN-γ-dependent and T-cells-independent pathways [49], negatively regulates VEGF-induced angiogenesis and markedly decrease blood vessel formation in the tumor tissues.

Apoptosis is an actively orchestrated programmed cell death pathway that serves to eliminate tumor and other harmful cells and to maintain tissue homoeostasis; thus it represents a strategic target for novel cancer therapies [50]. Human HCC, like other aggressive cancers, can easily escape form endogenous apoptosis and also has a notorious resistant to apoptosis induced by conventional treatment modalities. Thereby, development of new strategies to specifically and efficiently trigger apoptosis in HCC cells is a paramount therapeutic demand [12, 16]. In agreement, we herein observed that concurrent dual therapy with Ad-ΔB/TRAIL and Ad-ΔB/IL-12 had resulted in profound promotion of apoptosis in both HCC cells (as indicated by flow cytometry analysis) and in tumor tissues (as observed by TUNEL assay). In support with our findings, there is now a compelling body of evidence that TRAIL is a unique death ligand for induction of fulminant apoptotic effects in various human cancer cell types, including HCC, while showing only negligible effects on normal cells including normal hepatocytes [18–20, 51]. More importantly, earlier studies have reported that IL-12 sensitized HCC cells to TRAIL-induced apoptosis, and co-therapy with soluble IL-12 and TRAIL was synergistically interacted in apoptosis promotion and overcame TRAIL resistance in the human HCC cells isolated from HCC-bearing human patients or nude mice [27]. Next, to clarify the molecular mechanism whereby Ad-ΔB/TRAIL and Ad-ΔB/IL-12 were cooperatively interacted to promote apoptosis, we measured the expression of two common apoptosis-related proteins, caspase-3 and-8 [18], in the harvested liver tumor tissues of all treatment groups and our data revealed that there was a more significant upregulation of caspase-3 and -8 in Ad-ΔB/TRAIL plus Ad-ΔB/IL-12-treated tumor tissues, compared to therapy with Ad-ΔB. In short, our results may imply that the observed anti-HCC effect of Ad-ΔB/TRAIL plus Ad-ΔB/IL-12 combination therapy was, partly, mediated by activating caspase cascade apoptotic pathway in HCC cells and tumour tissues. In harmony, co-therapy with OAd-expressing TRAIL and another type of immunostimulant cytokine (IL-24) has been previously reported to be associated with potent activation in caspase pathway, particularly caspases-3 and -8, and apoptosis promotion in HCC [14].

## Conclusions

This preclinical study suggests for the first time that dual therapy with Ad-ΔB/TRAIL plus Ad-ΔB/IL-12 markedly suppresses human HCC by promoting anti-tumor apoptosis and immune activity, and also inhibits tumor angiogenesis and neovascularization. Because of therapeutic efficacy and easy accessibility, oncolytic adenovirus platform-co- delivering TRAIL and IL-12 genes might be a potential therapeutic strategy for treatment of human HCC. However, further studies are warranted to evaluate this therapeutic combination and also to explore its precise anti-tumor mechanisms.

## Additional file

Additional file 1: Figure S1. Histopathological assessment of liver tissue harvested at 3 days after systemic treatment of HCC-bearing mice with PBS, Ad-ΔB, or Ad-ΔB/TRAIL + Ad-ΔB/IL-12 (1 × 10^10^ VP, three times every other day). Each experiment was performed at least three times, and data shown are from representative experiments. (PPTX 227 kb)
