# Erratum to: Combined therapy with oncolytic adenoviruses encoding TRAIL and IL-12 genes markedly suppressed human hepatocellular carcinoma both in vitro and in an orthotopic transplanted mouse model

**DOI:** 10.1186/s13046-016-0365-4

**Published:** 2016-06-17

**Authors:** Adel Galal El-Shemi, Ahmad Mohammed Ashshi, Youjin Na, Yan Li, Mohammed Basalamah, Faisal Ahmad Al-Allaf, Eonju Oh, Bo-Kyeong Jung, Chae-Ok YUN

**Affiliations:** Department of Laboratory Medicine, Faculty of Applied Medical Sciences, Umm Al-Qura University, PO Box 7607, Holy Makkah, Saudi Arabia; Department of Bioengineering, College of Engineering, Hanyang University, 222 Wangsinmi-ro, Seongdong-gu, Seoul Korea; Graduate Program for Nanomedical Science, Yonsei University, Seoul, Korea; Department of Pathology, Faculty of Medicine, Umm Al-Qura University, Holy Makkah, Saudi Arabia; Science and Technology Unit & Department of Medical Genetics,Faculty of Medicine, Umm Al-Qura University, Holy Makkah, Saudi Arabia; Department of Pharmacology, Faculty of Medicine, Assiut University, Assiut, Egypt

## Erratum

Unfortunately, the original version of this article [1] contained an error. One of the subheadings within the “Methods” section read “Generation and purification of oncolytic adenoviruses expressing human TRAIL or human ING4 transgene”, but it should have read “Generation and purification of oncolytic adenoviruses expressing human TRAIL or human IL-12 transgene”.

This subheading has been also corrected in the original article.
