# Supplementary figures and images for: Combined therapy with oncolytic adenoviruses encoding TRAIL and IL-12 genes markedly suppressed human hepatocellular carcinoma both in vitro and in an orthotopic transplanted mouse model
Source: J Exp Clin Cancer Res. 2016 May 6;35:74. doi: 10.1186/s13046-016-0353-8 (PMC4859966; doi:10.1186/s13046-016-0353-8)

## Slide 1
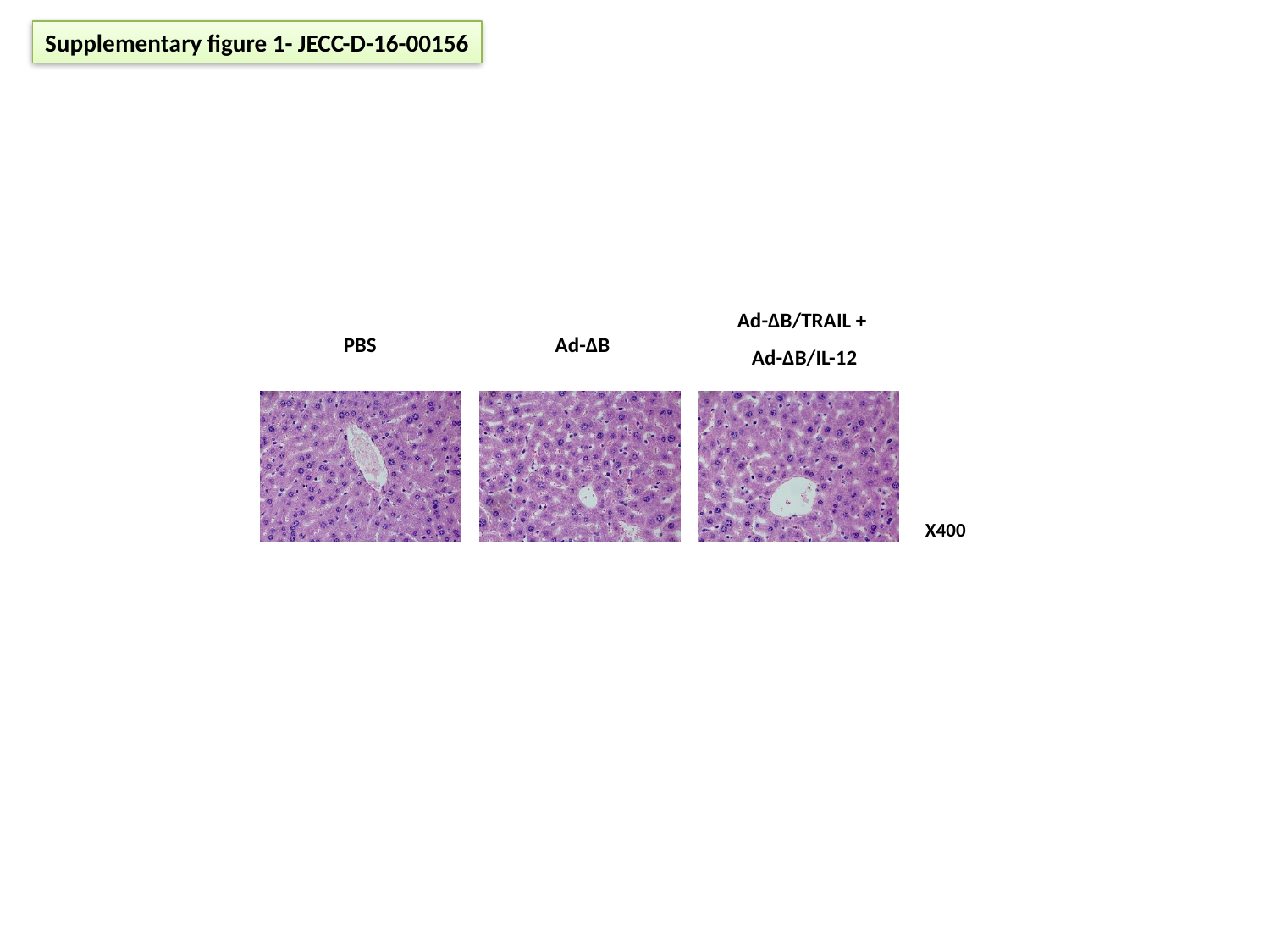

Supplementary figure 1- JECC-D-16-00156
PBS
Ad-ΔB
Ad-ΔB/TRAIL +
Ad-ΔB/IL-12
X400

Supplement: Additional file 1: Figure S1. — Histopathological assessment of liver tissue harvested at 3 days after systemic treatment of HCC-bearing mice with PBS, Ad-ΔB, or Ad-ΔB/TRAIL + Ad-ΔB/IL-12 (1 × 10^10^ VP, three times every other day). Each experiment was performed at least three times, and data shown are from representative experiments. (PPTX 227 kb) [file 13046_2016_353_MOESM1_ESM.pptx]
